# Discontinuation and switching of postpartum contraceptive methods over twelve months in Burkina Faso and the Democratic Republic of the Congo: a secondary analysis of the Yam Daabo trial

**DOI:** 10.1186/s40834-020-00137-2

**Published:** 2020-11-23

**Authors:** Abou Coulibaly, Tieba Millogo, Adama Baguiya, Nguyen Toan Tran, Rachel Yodi, Armando Seuc, Asa Cuzin-Kihl, Blandine Thieba, Sihem Landoulsi, James Kiarie, Désiré Mashinda Kulimba, Séni Kouanda, on behalf the study group, Nguyen Toan Tran, Wambi Maurice E. Yameogo, Félicité Langwana, Mary Eluned Gaffield, Armando Seuc, Asa Cuzin-Kihl, Seni Kouanda, Désiré Mashinda, Blandine Thieba, Rachel Yodi, Jean Nyandwe Kyloka, Tieba Millogo, Abou Coulibaly, Basele Bolangala, Souleymane Zan, Brigitte Kini, Sihem Landoulsi, James Kiarie

**Affiliations:** 1 Unité de Surveillance Démographique et de Santé (Kaya-HDSS), Institut de Recherche en Sciences de la Santé (IRSS) , 03 B.P. 7047, Ouagadougou, 03 Burkina Faso; 2 Ecole doctorale Sciences de la Santé, Université Joseph KI-ZERBO, 03 B.P. 7021, Ouagadougou, 03 Burkina Faso; 3 Institut Africain de la Santé Publique, 12 B.P, Ouagadougou, 199 Burkina Faso; 4 UNDP-UNFPA-UNICEF-WHO-World Bank Special Programme of Research, Development and Research Training in Human Reproduction (HRP), World Health Organization, Avenue Appia 20, 1211, 27 Genève, Switzerland; 5 Institute of Demography and Socioeconomics (IDESO), University of Geneva, Boulevard du Pont d’Arve 40, 1211 Geneva, Switzerland; 6 Australian Centre for Public and Population Health Research, Faculty of Health, University of Technology, PO Box 123, Sydney, NSW 2007 Australia; 7 Programme National de Santé de la Reproduction, Ministère de la Santé, Kinshasa, Democratic Republic of the Congo; 8 Unité de formation et de recherche en Sciences de la Santé, Université Joseph KI-ZERBO, 03 B.P. 7021, Ouagadougou, 03 Burkina Faso; 9 School of Public Health, University of Kinshasa, Kinshasa, Democratic Republic of the Congo

**Keywords:** Discontinuation, Switching, Modern contraception, Postpartum, Intervention

## Abstract

**Introduction:**

Women who use contraceptive methods sometimes stop early, use methods intermittently, or switched contraceptive methods. All these events (discontinuations and switching) contribute to the occurrence of unwanted and close pregnancies. This study aimed to explore contraceptive discontinuation and switching during the Yam-Daabo project to measure the effect of interventions on the continuation of contraceptive methods use.

**Methods:**

We conducted a secondary analysis of the Yam-Daabo trial data. We choose the discontinuation and switching of a modern contraceptive method as outcome measures. We performed a survival analysis using the Stata software package to estimate the effect of the interventions on contraceptive discontinuation. We also studied the main reasons for discontinuation and switching.

**Results:**

In total, 637 out of the 1120 women used at least one contraceptive method (of any type), with 267 women in the control and 370 in the intervention group. One hundred seventy-nine women of the control group used modern methods compared to 279 women of the intervention group with 24 and 32 who discontinued, respectively. We observed no statistically significant association between interventions and modern methods discontinuation and switching. However, modern methods’ discontinuation was higher in pills and injectables users than implants and IUDs users. The pooled data comparison showed that, in reference to the women who had not switched while using a modern method, the likelihood of switching to a less or equal effectiveness method among the women of the control group was 3.8(95% CI: 1.8–8.0) times the likelihood of switching to a less or equal effectiveness method among the women of the intervention group. And this excess was statistically significant (*p* < 0.001). The main reason for discontinuation and switching was method-related (141 over 199), followed by partner opposition with 20 women.

**Conclusion:**

The results of this study show no statistically significant association between interventions and modern methods discontinuation. Discontinuation is more related to the methods themselves than to any other factor. It is also essential to set up specific actions targeting women’s partners and influential people in the community to counter inhibiting beliefs.

**Trial registration:**

Pan African Clinical Trials Registry (PACTR201609001784334, https://pactr.samrc.ac.za/TrialDisplay.aspx?TrialID=1784).

## Background

Contraceptive use is very low in many developing countries. According to the United Nations Statistics Division (Africa Economic Commission), in 2012, Africa had the second-lowest contraceptive prevalence (44.3%) in the world, after Oceania (37.3%). More than 70% of African countries have a prevalence rate of less than 50%, which is why maternal mortality is high in Africa [1]. To improve the use of postpartum contraceptive methods, in 2015, we implemented a set of interventions in two African countries, Burkina Faso (BF) and the Democratic Republic of the Congo (DRC), and tested them in a cluster-randomized trial called Yam Daabo described previously [2, 3]. The main results showed a significant increase in the use of contraceptive methods among women receiving interventions compared to women in the control group in both countries [4, 5].

Contraceptive discontinuation and switching to a less effective method increase the risk of unwanted and closely-spaced pregnancies. In developing countries, short-term methods such as pills and injectables have discontinuation rates of about 40% at 12 months [6, 7]. The method-related concerns [8] and side-effects were the most commonly reported reasons for discontinuing these methods. Previous authors reported other factors as influencing contraceptive continuation. Among these factors, we have the use of a short-acting method, the desire for pregnancy within 2 years [9], little or no sexual relations [10], and other socio-economic and demographic factors such as age, marital status, income, mass media-exposure, and partner involvement in decision making, and service quality [11–13].

In the literature, few studies focused on the effect of family planning interventions on discontinuation and switching. We found three systematic reviews that concluded to a low level of evidence on the strategies’ effect to improve continuation of hormonal contraception [14–16]. Halpern et al., in 2013, reported that only three trials showed some benefit of strategies to improve adherence and continuation. Several studies included in the review had a small sample size, and six had a high number of lost to follow up [14]. The overall quality of evidence was considered moderate. For Mack et al., 2019, intensive counseling and reminders (with or without educational information) may be associated with the improved continuation of shorter-term hormonal contraceptive methods than usual family planning care. However, this should be interpreted with caution due to the low certainty of the evidence [15]. Cavallaro et al., in 2013, conducted a systematic review of the strategies to improve adherence and acceptability of hormonal methods of contraception. A total of 63 publications corresponding to 61 studies met their inclusion criteria. There was substantial heterogeneity in study settings, interventions, and outcome measures. Interventions targeting women initiating a method (including structured counseling on side effects) tended to show positive contraceptive continuation effects. In contrast, most studies on providers’ training and decision-making tools for method choice did not find evidence of an effect [16].

After showing that family planning interventions had low evidence on contraceptive discontinuation, Halpern et al., in 2013, suggested that high-quality randomized controlled trials, with adequate power, and well-designed interventions, could help to identify ways to improve women’s adherence to hormonal contraceptive methods [14]. Furthermore, Cavallaro et al., mentioned the need to improve the reporting of studies and develop and evaluate novel interventions in different settings. Therefore, this secondary analysis of Yam Daabo data which had been implemented in a predominantly rural context for Burkina Faso and urban context for the DR Congo, can contribute to addressing these evidence gaps. So, we aimed to explore contraceptive discontinuation and switching among the postpartum intervention women. We assessed if contraceptive users in the intervention group continued their choice method for a longer time or switched to an equally or more effective method, compared to those in the control group, during the first 12 months postpartum. In this study, we also aimed to explore the reasons for contraceptive switching among postpartum women.

## Methods

We performed a secondary analysis of the Yam Daabo study, which was a two-group, multi-intervention, single-blinded, cluster-randomized controlled trial with health centres as the randomization units. It was a study that involved two countries: Burkina Faso and the Demographic Republic of the Congo. Health centres in each country were randomized into two groups: intervention and control. The intervention group’s health centres offered a set of six postpartum family planning (PPFP) interventions that were identified as solutions to the barriers identified during the planning phase of the project [3]. The control group health centres provided the usual care of PPFP. The study had the statistical power to detect a 15-point difference between the intervention and control groups in terms of the proportion of women adopting an effective PPFP method at 6-month. In each country, 8 health centres were selected (4 intervention and 4 controls) for the study, and, taking into account the loss to follow-up, each centre had to include 70 women (refer to the published protocol [2]).

The project interventions can be categorized into two broad groups: the supply-side interventions were the improvement of the availability of the PPFP services 7 days a week and training/updating the clinical skills of health providers on the PPFP, including capacity building support supervision for service providers; and the demand-side interventions such as the PPFP counselling tool (new intervention tool taking into account all of World Health Organization’s (WHO) new recommendations for offering FP services), appointment cards for women, and invitation letters for partners [3]. We included a total of 1147 women in both countries, and 1120 women had follow-up data.

The WHO Research Ethics Review Committee approved the trial as well as the ethics committee for health research in Burkina Faso and the School of Public Health ethics committee in DR Congo. Moreover, the trial was registered in the Pan African Clinical Trials Registry (PACTR201609001784334).

For the analysis of switching of the different methods, we adopted the classification of Trussels et al., which considered the switching between four groups (from the less effective to the more effective) [17] which are:

- First group: spermicide (correct use: at every sex), abstain, collier, other methods
- Second group: condoms, diaphragm, sponge, withdrawal (correct use: at every sex)
- Third group: Injections (repeat injections on time), Lactational Amenorrhea Method (LAM) until 6 months, Pills, patch, ring
- Fourth group: vasectomy (with the use of another method for the first 3 months), implant, Intrauterine device (IUD), and female sterilization

We had no data on adherence to the method. Hence we assumed that women used them correctly (especially for pills and injectables).

Discontinuation is defined as starting modern contraceptive use within 12 months postpartum and then stopping for any reason while still at risk of unintended pregnancy. If the woman stops using the second after switching the first one, the use duration is that of the second method.

An episode of switching occurred when a woman, using a given contraceptive method, change for any reason to another contraceptive method. We excluded the women who stopped the first method because we could not classify them according to the chosen classification. So, we only considered those who used pills or IUDs or injectables or implants at least once during the follow-up for discontinuation and switching analysis. We did not consider the condom because it was used in combination with other methods (17 women). LAM was also not considered because it is ineffective after 6 months postpartum and, therefore, necessarily leads to another more effective method.

We chose the discontinuation (for modern contraceptive methods) and the switching (for any method) as outcome measures. To compute the durations of the two outcomes, we listed different situations:

- Women started using a method not classified in this analysis (abstain, collier, condoms, diaphragm, sponge, withdrawal, LAM until 6 months, and other methods) and haven’t used another. We ignored these women in the statistical analysis.
- Women started by using a method not classified in this analysis and switched to pills, injectables, implants or IUDs. The duration of use was the time between the second method initiation and the end of the study (or the last follow-up date if the woman was lost-to-follow-up).
- Women started by using pills, injectables, implants, or IUDs and switched to pills, injectables, implants, or IUDs and then stopped the second method. The duration of use was the duration of the use of the second method.
- Women started contraception with pills, injectables, implants, or IUDs and switched to a method not classified in this analysis (abstain, collier, condoms, diaphragm, sponge, withdrawal, LAM until 6 months, and other methods) and then stopped the second method. The duration of use was the duration of the use of the first method.

We used survival analysis to estimate the effect of postpartum interventions on contraceptive discontinuation and switching. This technique allowed to include censored episodes in the estimation procedures. In this study, we defined an episode as a period of uninterrupted use of a contraceptive method that may or may not has ended. If the episode ended without switching to another method, then it was discontinuation. One woman may report several episodes of contraceptive use. If another method was used after the first episode, then it was switching. The woman was right-censored if she started a method and did not stop it for the rest of the follow-up. She was left-censored if she started a method other than those excluded in the operational definitions given above.

The statistical significance and the effect of postpartum interventions on each outcome of interest were assessed using multivariate regression modeling. We performed maximum likelihood estimation for parametric regression survival-time models. 95% confidence interval (CI) and *P*-value less or equal to 0.05 were set to determine the statistical significance level.

After comparing the different models using the Akaike’s Information Criteria (AIC), the best model was the Weibull survival distribution model (*streg* Stata’s command). Therefore, it was selected to estimate the effect of the interventions on contraceptive discontinuation. We opted for a hazard ratio (HR) estimate for our exposure variable and the covariables.

Bivariate analysis with cluster effect correction was conducted initially to measure the possible association between interventions and the discontinuation or switching of the contraceptive methods, with primary health centres as clusters. Then, we adjusted the estimates by introducing into the model the type of method used. We analyzed each country separately and pooled both countries’ data because they are different settings (urban in the DRC, primarily rural in Burkina Faso).

For switching, we used a multinomial logit model to estimate the effect of interventions on switching to a less or equal effectiveness method or a more effective method. The reference group was that of the women who did not switch the first method.

We also reported the reasons of switching by method type on the one hand and also by the interventions group on the other hand.

## Results

In total, 637 (56.8%) out of the 1120 women used at least one contraceptive method (of any type), with 47.8% (267/558) of users in the control group and 65.8% (370/562) in the intervention group.

### Discontinuation of modern methods

Hormonal methods (pills, injectables, and implants) and intrauterine devices were used by 179 women of the control group with 24 discontinuations compared to 279 women of the intervention group with 32 discontinuations. The incidence rate of discontinuation of hormonal contraceptives was 6.91 in the control group compared to 5.14 per 10,000 women-days in the intervention group for the pooled data. The country-specific and pooled results are expressed in Table 1.

**Table 1:** Episodes of contraceptive use (pills, injectables, implants, and intra-uterine devices) by study group and country

	Burkina Faso		DR Congo		Pooled data	
	Control	Intervention	Control	Intervention	Control	Intervention
Number of episodes	99	181	80	98	179	279
Total time at risk (days)	22,747	40,839	11,981	21,418	34,728	62,257
Number of discontinuations	17	28	7	4	24	32
Incidence rate (per 10,000 women-days)	7.474	6.856	5.843	1.868	6.911	5.140
Mean time of use (days)	230	226	150	219	194	223
Median time	251	252	140	235	193	249
Hazard ratio	0.92 (0.43–1.96)		0.32 (0.05–2.00)		0.69 (0.32–1.50)	

No statistically significant difference was observed between study groups regarding modern method discontinuation in either the bivariate or multivariate analyses. However, the discontinuation of modern methods was explained mainly by the type of method used. Compared to users of long-acting and reversible contraceptives (implants and IUD), women using injectables and pills were 13 and 10 times, respectively, more likely to discontinue its use during the first postpartum 12 months (95% CI = 4.5–38.0 and 3.7–28.7, respectively). All the other covariables, such as the number of pregnancies, abortion, living children, women’s education, and women’s occupation, showed no significant association with our variable of interest (Table 2).

**Table 2:** Univariate and multivariate analysis of interventions’ effects on method discontinuation

	Crude Hazard Ratio (95% CI)	p	Adjusted Hazard Ratio (95% CI)	p
Group				
Control	Ref		Ref	
Intervention	0.7 (0.3–1.5)	0.351	0.9 (0.4–1.9)	0.701
Method type				
Injectables	13.3 (4.8–36.9)	0.000	13.0 (4.5–38.0)	0.000
Pills	10.5 (3.8–28.8)	0.000	10.3 (3.7–28.7)	0.000
Implants/IUD	Ref		Ref	

### Switching of contraceptives methods

Table 3 compares the method switching risks between the control and intervention groups in DR Congo, Burkina Faso, and both countries combined.

**Table 3:** Multinomial logit estimates, comparing risks of switching between study group by country

	Burkina Faso			DRC			Pooled Data		
	n(%)	HR(95%CI)	p	n(%)	HR(95%CI)	p	n(%)	HR(95%CI)	p
No switching									
Control	74 (90.2)			50 (48.5)			124 (67.0)		
Intervention	120 (72.7)			88 (88.9)			208 (78.8)		
Switching to less or equally effective methods									
Control	2 (2.4)	0.5 (0.1–2.7)	0.458	23 (22.3)	8.1 (2.9–22.6)	0.000	25 (13.5)	3.8 (1.8–8.0)	0.000
Intervention	6 (3.6)	Ref		5 (5.1)	Ref		11 (4.2)	Ref	
Switching to more effective methods									
Control	6 (7.3)	0.2 (0.1–0.6)	0.003	30 (29.1)	8.8 (3.4–22.6)	0.000	36 (19.5)	1.3 (0.8–2.2)	0.241
Intervention	39 (23.6)	Ref		6 (6.1)	Ref		45 (17.0)	Ref	

In the DR Congo, in reference to the women who had not switched while using a modern method, the likelihood of switching to a less or equally effective method among women of the control group was 8.1(95% CI: 2.9–22.6) times higher than that among women of the intervention group (*p* < 0.001). This result is similar to that comparing the likelihood of switching to a more effective method among the women of the control group to the likelihood of switching to a more effective method among the women of the intervention group (RRR = 8.8(95% CI: 3.4–22.6)) in reference to the women who had not switched while using a modern method.

In Burkina, in reference to the women who had not switched while using a modern method, none difference was found between the likelihood of switching to a less or equally effective method among women of the control group and that among women of the intervention group (*p* = 0.458). But, in reference to the women who had not switched while using a modern method, we found a reduction of 80% in the likelihood of switching to a more effective method among women in the control group compared to that among women of the intervention group (RRR = 0.2(95% CI: (0.1–0.6)). In other words, the women in Burkina’s intervention group likely switched to a more effective method than women in the control group.

The pooled data comparison showed, in reference to the women who had not switched while using a modern method, the likelihood of switching to a less or equally effective method among the women of the control group was 3.8(95% CI: 1.8–8.0) times higher than that the likelihood of switching to a less or equal effectiveness method among the women of the intervention group. And this excess was statistically significant (*p* < 0.001).

### Main reasons for first adopted method discontinuation or switching

Out of 199 women who switched, 141were interrupted due to a method-related reason (mainly lactational amenorrhea method). The second and third reason was partner opposition and unknown reason in respectively 20 and 17 cases. Table 4 shows these results.

**Table 4:** Main reasons for discontinuation or switching of first adopted methods by contraceptive type

	LAM	Injectable	Daily Pill	Implant/IUD	Condom	Other methods	Total
	n(%)	n(%)	n(%)	n(%)	n(%)	n(%)	n(%)
Method-related reason^a^	64 (45.4)	20 (14.2)	12 (8.5)	5 (3.5)	14 (9.9)	26 (18.4)	141 (100.0)
Partner opposition	0 (0.0)	3 (15.0)	2 (10.0)	0 (0.0)	6 (30.0)	9 (45)	20 (100.0)
Unknown	1 (5.9)	6 (35.3)	1 (5.9)	1 (5.9)	6 (35.3)	2 (11.8)	17 (100.0)
Reduced need^b^	0 (0.0)	5 (62.5)	2 (25.0)	0 (0.0)	0 (0.0)	1 (12.5)	8 (100.0)
Desire for a child	0 (0.0)	2 (40.0)	1 (20.0)	1 (20.0)	0 (0.0)	1 (20.0)	5 (100.0)
Pregnancy	0 (0.0)	0 (0.0)	2 (50.0)	1 (25.0)	1 (25.0)	0 (0.0)	4 (100.0)
Financial problem	0 (0.0)	4 (100.0)	0 (0.0)	0 (0.0)	0 (0.0)	0 (0.0)	4 (100.0)
**Total**	**65 (32.7)**	**40 (20.1)**	**20 (10.1)**	**8 (4.0)**	**27 (13.6)**	**39 (19.6)**	**199 (100.0)**

^a^: Fear of side effects, side effects experienced, switching to a more effective method, switching to a more convenient method, method ineffective, noncompliance

^b^: Reduced need included partner traveling and no partner (deceased or separated)

The proportions of the main reasons were not different between the two groups of women (significance not statistically evaluated) excepted for the partner opposition. Indeed, in the control group, 14 women (16.3%) stopped using their first method or changed it because of their partner disapproval against 6 (5.3%) in the intervention group. Table 5 expressed all these results.

**Table 5:** Main reasons for first adopted method discontinuation or switching by study group

	Control	Intervention	Total
	n(%)	n(%)	n(%)
Method-related reason	58 (67.4)	83 (73.5)	141 (70.9)
Partner opposition	14 (16.3)	6 (5.3)	20 (10.1)
Unknown	7 (8.1)	10 (8.8)	17 (8.5)
Reduced need	1 (1.2)	7 (6.2)	8 (4.0)
Pregnancy	0 (0.0)	4 (3.5)	4 (2.0)
Financial problem	4 (4.7)	0 (0.0)	4 (2.0)
Desire for a child	2 (2.3)	3 (2.7)	5 (2.5)
**Total**	**86 (100.0)**	**113 (100.0)**	**199 (100.0)**

Regarding the discontinuation among women who were using injectables, 13 women (total of 20) who discontinued the use gave the method-related reason to explain it in the control group against seven women (total of 20 also) in the intervention group (results not shown in the table).

## Discussion

At the end of this analysis, we noted that postpartum interventions (Yam Daabo project) did not significantly affect modern contraceptive method discontinuation. We also reported different results between the two countries in method switching. Indeed, while women in the intervention group in Burkina Faso switched contraceptive methods less than those in the control group, in the DRC, the situation was the opposite (more frequent method switching in the intervention group). The main reason for the first method switching was method-related reasons, although some women also cited partner opposition as a reason for discontinuation or switching contraceptive methods.

### Effect of PP interventions on contraceptive methods discontinuation and associated factors

The results showed no effect of PP (Yam Daabo) interventions on contraceptive methods discontinuation. Instead, these events were associated with the type of contraceptive methods used. Weldemariam et al. showed that the method related problems were found to contribute to more than half of the contraceptive use discontinuation by studying the reasons and multilevel factors associated with unscheduled contraceptive use discontinuation in Ethiopia [8]. In their study, they found that IUD and implant discontinuation rates were lowest compared to others. In our research, we had the same finding. Indeed, compared to implants or IUDs, we noticed many discontinuations among women using pills and injectables, which could be explained by the complexity of their intake (daily intake). The woman may either forget to take the pull several times or not take the pill at the specified times. These events can lead to a switch to a more practical method, especially to long-acting methods (implant and IUD), and to injectables (to a lesser extent). These results, obtained in the multivariate analysis, are consistent with women’s reasons to explain the discontinuation of the methods. According to them, the method-related issues were the main reason for discontinuation of the first method, with 141 episodes stopped over 199 (70.8%). The periodicity of injectable renewal exposes women to forgetfulness in the same way as pills. So, this can lead to switching or discontinuation. The other reason for the low likelihood of women using a long-acting method to switch or stop is that these methods are administered by qualified staff, and discontinuing or switching also requires the same staff type. Women who return to interrupt her long-acting method could receive explanations (discussions on the reasons for the discontinuation) from health workers. They may convince her not to stop the method; this is unlike the pill or injectables, for which a woman does not need to see a health worker to stop the method. All of these reasons explain why, in almost all studies, such as the Casey et al. ‘s study in the DRC (86.1% versus 78.0%), the rate of continuation of methods is higher in users of long-acting methods (implants and IUDs) than in users of short-acting methods (pills and injectables) [9]. In Senegal, in 2015, a study of 6927 women of childbearing age living in six urban sites showed that implants had the lowest 12-month discontinuation rate (6.3%), followed by intrauterine devices (IUDs) (18.4%). Higher rates were observed for injectable contraceptives (32.7%), pills (38%), and condoms (62.9%) [11]. Similar results were reported by the Diedrich’s study, which, after adjustment, showed a risk of discontinuation that was three times higher among users of other methods compared to long-acting methods users (HRa =3.08, 95% CI = 2.80–3.39) [18].

In this study, discontinuation and switching were more frequently observed among women using LAM. Indeed, this method is ineffective after 6 months postpartum, and the women who were using are supposed to know that they must change to another method after 6 months postpartum. The counseling made with our new tool could have contributed to reassure the women for these switching. Also, regarding the data on discontinuation among injectables users, we noted that the control group had many women who gave method-related reasons to discontinue the method compared to the intervention group, which could also be explained by the counseling tool. Indeed, before adopting the given method, health workers had to explain the side effects to a woman so that she was prepared to accept any symptom that she might experience while using the method. Health care providers might be updated on contraceptives side-effects, indications, contraindications, and mechanisms of action.

### Effect of PP (yam Daabo) interventions on contraceptive methods switching

Regarding the switching of methods, we noted that switching of methods varied according to the study group. Being part of the control group was associated with a decreased likelihood of switching to a less or equal effectiveness method, especially in the RD Congo. This shows a beneficial effect of PP interventions on switching.

### Reason of contraception discontinuation or switching

Lastly, regarding the reason of contraception discontinuation or switching, some women did not indicate their reasons for the discontinuation and switching of methods (17 women in total, similar repartition between the study’s group). This lack of clear reason raises questions, especially in an African context, marked by misconceptions about contraception, as shown in this trial’s first phase [19]. A literature review by Blackstone et al., published in 2017 regarding factors influencing contraceptive use in sub-Saharan Africa between 2005 and 2015 showed that negative factors prohibiting or reducing contraceptive use were women’s misconceptions of contraceptive side–effects, male partner disapproval, and social/cultural norms surrounding fertility. Positive factors included education, employment, and communication with male partners [20]. So, one of the common reasons for discontinuation given by women is their partner’s opposition. This has already been reported in several studies [19–23] as one of the inhibitors for contraception use by women in general and especially by married women. The opposition from partners calls for more targeted action towards men to gain a better commitment from them to facilitate the use of contraceptive methods by women. In particular, family planning is part of women’s rights, and husbands should not prevent them from adopting their choice method. In this study, we noted a different distribution of women who gave their partner’s opposition as the reason for discontinuation even if the statistical significance was not explored (in the control group, 14 women (16.3%) against 6(5.3%) in the intervention group).

### Limitations

For the analysis of switching, we did not consider the time for which women used the first modern methods if they used more one modern contraceptive method.

## Conclusion

We noted that postpartum interventions did not significantly affect modern contraceptive method discontinuation. Discontinuation is much more related to the methods themselves than to any other factor. It is also essential to set up specific actions targeting women’s partners and influential people in the community to counter inhibiting beliefs. The capacity to build on health care providers’ knowledge of the indications, contraindications and mechanisms of action of the various contraceptive methods could also improve women’s adherence.

## Data Availability

Requests for the anonymised, coded trial data can be made to the Department of Reproductive Health and Research, World Health Organization (reproductivehealth@who.int). Data sharing is subject to WHO data sharing policies and data use agreements with the participating research centres.

## Abbreviations

BF: Burkina Faso
DRC: Democratic Republic of the Congo
AIC: Akaike’s Information Criteria
CI: Confidence interval
HR: Hazard ratio
HRa: Adjusted Hazard ratio
IUD: Intrauterine device
LAM: Lactational Amenorrhea Method
PP: Postpartum
PPFP: Postpartum family planning
WHO: World Health Organization
